# 

**Published:** 1869-12

**Authors:** 


### Books and Pamphlets Received.

Transactions of the American Medical Association. Volume Twenty. 1869. Philadelphia.

A Practical Treatise on the Diseases of Children. By Alfred Vogel, M.D., Professor of Clinical Medicine in the University of Dorpat, Russia. Translated and edited by H. Raphael, M. D. From the Fourth German Edition. D. Appleton & Co., New York. For sale by Breed & Lent.

A Treatise on Intra-ocular Tumors. From Clinical observations and Anatomical investigations. With Lithographic Plates and Figures. By H. Knapp, late Professor of Ophthalmology and Surgeon to the Ophthalmic Hospital in Heidelburg. Translated by L. Cole, M. D., of Chicago. Wm. Wood Co., New York. For sale by Breed & Lent.

On the Wasting Diseases of Infants and Children. By Eustace Smith, M. D., London. Member of the Royal College of Physicians; Physician extraordinary to the King of the Belgians, etc., etc. Henry C. Lea, Philadelphia. For sale by Breed & Lent.

Annual Report of the Surgeon General of the United States Army. 1869.

Transactions of the Medical Society of the State of West Virginia. Wheeling.

The Nomenclature of Diseases. By a Joint Committee appointed by the Royal College of Physicians of London. Printed by order of the American Medical Association.

Annual Report of the Board of Regents of the Smithsonian Institution for the year 1868.

Annual Announcement of the Medical School of Boudoin College, Brunswick, Maine.

Report on the Best methods of Treatment for different forms of Cleft Palate. By Wm. R. Whitehead, M. D., New York.

A Review of the Last Illness of Dr. Alden March. A Criticism on the Management of his case. By Chas. A. Robertson, A. M., M. D., Ophthalmic and Aural Surgeon of the Albany Hospital, etc.

Nocturnal Enuresis and Incontinence of Urine. By Frederick G. Snelling, M. D.

The History of Nine Cases of Ovariotomy. By T. Gaillard Thomas, M. D., Professor of Obstetrics, and Diseases of Women and Children, in the College of Physicians and Surgeons, New York. D. Appleton & Co.

Eulogium on Thomas C. Brinsmade, M. D. By Geo. H. Hubbard, A. M., M.D.

Biographical Sketch of the late A. B. Shipman, M. D., of Syracuse, N. Y. By H. O. Jewett, M. D., Cortland, N. Y.

Vesico-vaginal Fistule: and its successful Treatment by the Button Suture. By Nathaniel Bozeman, M. D., New York.

Three Cases of Lead Palsy from the use of a Cosmetic called "Laird's Bloom of Youth." By Lewis A. Sayre, M. D., New York.

Twenty-fourth Annual Report of the Managers of Mass. Charitable Eye and Ear Infirmary. November, 1869.

The Pathology of Bright's Disease. By Wm. B. Lewis, M. D., Lecturer on Renal Pathology in the Medical Department of the University of New York.

The Practitioner: a monthly journal of Therapeutics. By Francis E. Anstie, M. D. Kelly, Piet & Co., Baltimore.

Wood's Household Magazine. S. S. Wood, Newburgh, N. Y.

The American Stock Journal, Parkersburgh, Chester Co., Pa.

The Old Franklin Almanac for 1870. A. Winch, Philadelphia.

The Printers Bulletin Boston.

The Scientific American.

The Atlantic Monthly.

